# CD38-directed CAR-T cell therapy: a novel immunotherapy strategy for relapsed acute myeloid leukemia after allogeneic hematopoietic stem cell transplantation

**DOI:** 10.1186/s13045-021-01092-4

**Published:** 2021-05-25

**Authors:** Qingya Cui, Chongsheng Qian, Nan Xu, Liqing Kang, Haiping Dai, Wei Cui, Baoquan Song, Jia Yin, Zheng Li, Xiaming Zhu, Changju Qu, Tianhui Liu, Wenhong Shen, Mingqing Zhu, Lei Yu, Depei Wu, Xiaowen Tang

**Affiliations:** 1 National Clinical Research Center for Hematologic Diseases, Jiangsu Institute of Hematology, The First Affiliated Hospital of Soochow University, Suzhou, 215006 China; 2 Institute of Blood and Marrow Transplantation, Collaborative Innovation Center of Hematology, Soochow University, Suzhou, 215123 China; 3 School of Chemistry and Molecular Engineering, East China Normal University, Shanghai, 200062 China; 4 Shanghai Unicar-Therapy Bio-Medicine Technology Co., Ltd., Shanghai, 201203 China; 5 Jiangsu Institute of Hematology, The First Affiliated Hospital of Soochow University, Institute of Blood and Marrow Transplantation of Soochow University, Suzhou, 215000 China

**Keywords:** Chimeric antigen receptor T cells, CAR-T-38, Relapsed acute myeloid leukemia, Allogeneic hematopoietic stem cell transplantation, Cytokine release syndrome

## Abstract

Allogeneic hematopoietic stem cell transplantation (allo-HSCT) is a potentially curative treatment for acute myeloid leukemia (AML). However, most patients experience relapse after allo-HSCT, with a poor prognosis, and treatment options are limited. The lack of an ideal targetable antigen is a major obstacle for treating patients with relapsed AML. CD38 is known to be expressed on most AML and myeloma cells, and its lack of expression on hematopoietic stem cells (HSCs) renders it a potential therapeutic target for relapsed AML. To investigate the clinical therapeutic efficacy and safety of CD38-targeted chimeric antigen receptor T (CAR-T-38) cells, we enrolled 6 AML patients who experienced relapse post-allo-HSCT (clinicaltrials.gov: NCT04351022). Prior to CAR-T-38 treatment, the blasts in the bone marrow of these patients exhibited a median of 95% (92–99%) CD38 positivity. Four weeks after the initial infusion of CAR-T-38 cells, four of six (66.7%) patients achieved complete remission (CR) or CR with incomplete count recovery (CRi); the median CR or CRi time was 191 (range 117–261) days. The cumulative relapse rate at 6 months was 50%. The median overall survival (OS) and leukemia-free survival (LFS) times were 7.9 and 6.4 months, respectively. One case relapsed 117 days after the first CAR-T-38 cell infusion, with remission achieved after the second CAR-T-38 cell infusion. All six patients experienced clinically manageable side effects. In addition, multiparameter flow cytometry (FCM) revealed that CAR-T-38 cells eliminated CD38 positive blasts without off-target effects on monocytes and lymphocytes. Although this prospective study has a limited number of cases and a relatively short follow-up time, our preliminary data highlight the clinical utility and safety of CAR-T-38 cell therapy in treating relapsed AML post-allo-HSCT.

To the Editor,

Acute myeloid leukemia (AML) patients who experience relapse after allogeneic hematopoietic stem cell transplantation (allo-HSCT) have a dismal prognosis, with a one-year overall survival (OS) rate of only 20% [1, 2]. Recently, chimeric antigen receptor T (CAR-T) cell therapy has emerged as one of the most promising therapeutic approaches for those with relapsed/refractory B-cell acute lymphoblastic leukemia (r/r B-ALL) [3, 4]. Although numerous tumor antigens, such as CD33, CD123 and CLL1, have been explored as potential target antigens for AML treatment in the past few decades [5–7], CAR-T cell therapy in AML remains challenging due to the lack of an ideal specific antigen target and the risk of fatal “off-tumor, on-target” side effects [8].

CD38 is known to be expressed on most AML blast cells or plasma cells in multiple myeloma but not on healthy hematopoietic stem cells (HSCs) [9]. Studies of daratumumab monotherapy targeting CD38 report encouraging clinical efficacy, favorable safety profiles and mild hematologic toxicities in multiple myeloma patients [10]. To investigate whether a similar immunotherapeutic approach can be applied for the treatment of AML, we selected the same CD38 epitope (CD38 scFv) as daratumumab for our CD38-CAR-T cells (CAR-T-38) (Fig. 1a). Theoretically, CAR-T-38 cells should exhibit anticancer effects and safety profiles similar to those of daratumumab; moreover, CAR-T-38 cells possess innovative “live-drug” functionality. In this prospective study (NCT04351022), we evaluated the clinical therapeutic efficacy and safety of CAR-T-38 cell therapy in six patients with relapsed AML post-allo-HSCT. None of the cases responded to multiple lines of salvage treatments, and all had CD38-positive blasts in their bone marrow at the time of relapse. The patients’ clinical characteristics are summarized in Table 1. For the initial tumor-reduction chemotherapy (Fig. 1b), only patient 1 achieved blast reduction from 15.5% to 5%, whereas the remaining five showed no response. All patients were pretreated with fludarabine and cyclophosphamide (FC) regimens prior to CAR-T-38 immunotherapy. CAR-T-38 cells (4 from autologous and 2 from donors) at a median dose of 8.05 (6.1–10) × 10 [6] cells per kilogram of body weight were administered after the FC regimens. The median marrow donor chimerism recovered from 46.3% (26.6–66.4%) to 92.5% (58.9–98.1%) at one week and to 97.5% (13.5–98.4%) at two weeks post-CAR-T-38 infusion (Fig. 1c). Moreover, at four weeks after CAR-T-38 infusion, 66.7% of patients (4/6; patients 1, 2, 4 and 5) achieved complete remission (CR) (including 1 with CR and 3 with CR with incomplete count recovery (CRi)) and full donor chimerism. In contrast, patient 6 exhibited a 44% reduction in bone marrow blasts; patient 3 showed no response (NR). Interestingly, patient 4 experienced relapse 117 days after the first CAR-T-38 infusion but did achieve remission after a second CAR-T-38 treatment (Fig. 1d). OS was calculated from the date of CAR-T-38 infusion to the date of death. Leukemia-free survival (LFS) was calculated from the date of CR or CRi post-CAR-T-38 infusion to the date of relapse or death or the last follow-up. The 6-month OS and LFS rates were both 50%, and the median OS and LFS were 7.9 and 6.4 months, respectively (Fig. 1d). Multiparameter flow cytometry (FCM) revealed that CD38-positive blasts were effectively eliminated after CAR-T-38 infusion (Fig. 1e). However, CD38-positive granulocytes and monocytes increased gradually after CAR-T-38 infusion (Fig. 1f, g).

**Fig. 1:**
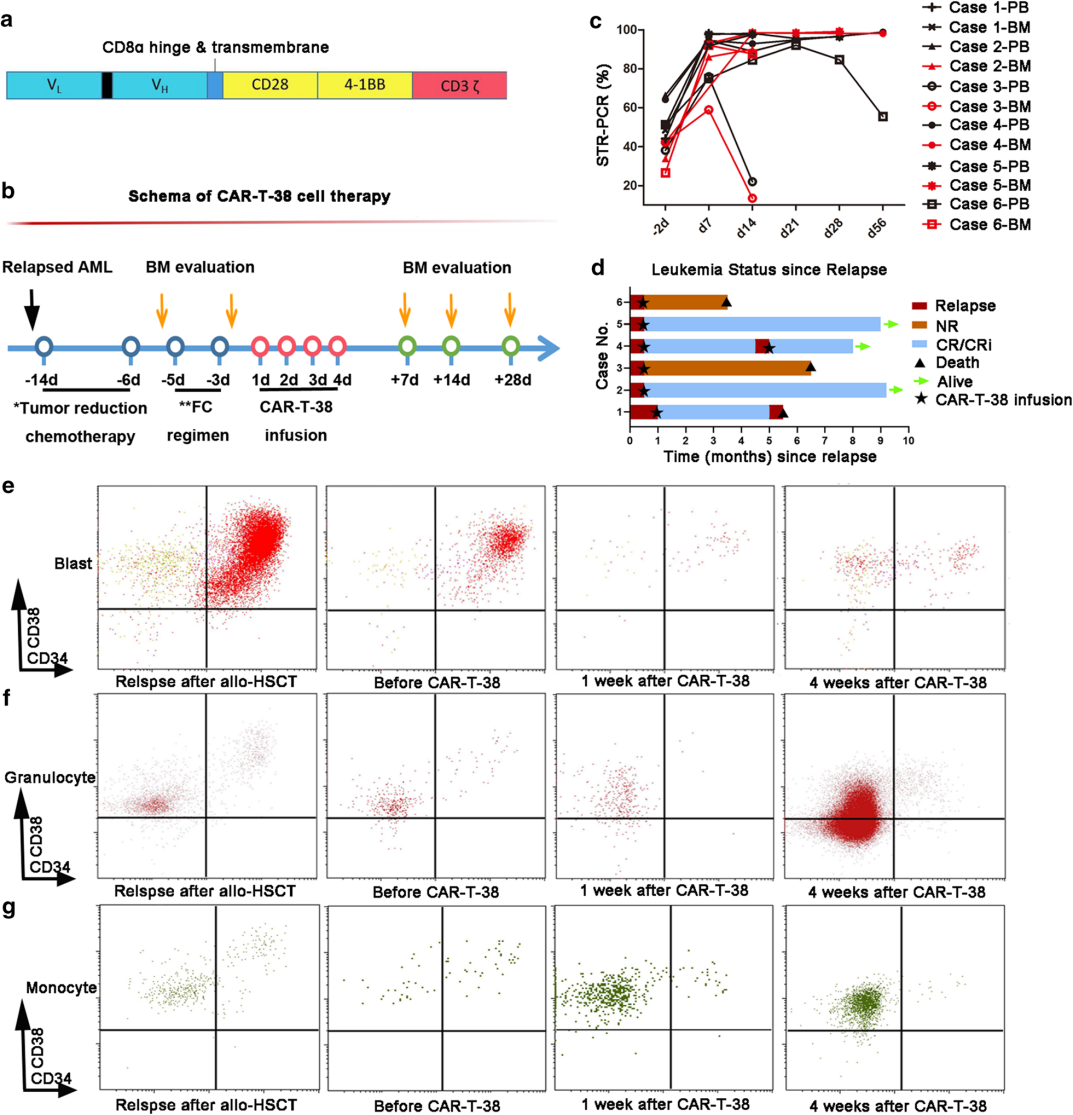
CAR-T-38 therapy regimen and treatment response among 6 relapsed AML patients post-allo-HSCT. **a** Schematic of the CAR-T-38 structure. **b** Schematic of the CAR-T-38 therapy regimen (* and ** supplementary file). **c** Variation in donor chimerism in the bone marrow (BM) and peripheral blood (PB) was measured by short tandem repeat-polymerase chain reaction (STR-PCR). **d** Patients 1, 2, 4, and 5 achieved complete remission (CR) or CR with incomplete count recovery (CRi) at 4 weeks after CAR-T-38 infusion. Patients 1 and 4 relapsed within six months. Patient 4 experienced relapse 117 days after the first CAR-T-38 infusion but exhibited remission after the second CAR-T-38 treatment. The 6-month OS and LFS rates were both 50%. CD38-positive blasts were reduced, and CD38-positive granulocytes and monocytes gradually increased at 1 and 4 weeks after CAR-T-38 infusion. **e** The CD38-positive blast population is shown in the upper right (red) of the CD34/CD38 plot. **f**, **g** CD38-positive granulocytes and monocytes, based on forward versus side scatter and CD38 expression, are shown in the upper left (crimson) and the lower left (green), respectively

**Table 1:** Clinical characteristics of patients

Patient characteristics	N = 6 (%)
Median age (range) years	34.5 (7–52)
Female sex	1 (16.7%)
ECOG performance status ≤ 2	6 (100%)
*Disease*	
AML	5 (83.3%)
AML evolving from MDS	1 (16.7%)
Median percentage of CD38 positive blasts (%)	95% (92–99%)
*Allo-HSCT donor*	
Unrelated donor (10/10)	2 (33.3%)
Haplo donor (5/10)	4 (66.7%)
*Conditioning regimen*	
Modified BuCy	5 (83.3%)
Decitabine + TBI-Cy	1 (16.7%)
Median time to recurrence post-allo-HSCT (months)	7.5 (5–17)
*Treatment for relapsed AML post-allo-HSCT*	
Withdrawal of immunosuppression	5 (83.3%)
Donor lymphocyte infusion	2 (33.3%)
Azacitidine + Venetoclax	2 (33.3%)
Lenalidomide	1 (16.7%)
Interferon-α	1 (16.7%)
*Tumor reduction chemotherapy before CAR-T-38 infusion*	
Decitabine + HAAG	5 (83.3%)
Decitabine + EAAG	1 (16.7%)
*Source of CAR-T-38*	
Autologous	4 (66.7%)
Donor	2 (33.3%)
Median CAR-T-38 dose (range)	8.05 (6.1–10) × 10^6^/kg
*Treatment response*	
1 week after CAR-T-38 infusion	2 CRi (33.3%)
2 weeks after CAR-T-38 infusion	4 CRi (66.7%)
4 weeks after CAR-T-38 infusion	4 (3 CR and 1 CRi) (66.7%)
6-month cumulative recurrence	2 (50%)
*Adverse events*	
GvHD	0 (0.0%)
CRS grade 1–2	5 (83.3%)
CRS grade 3 (hematological toxicity)	1 (16.7%)
ICANS	0 (0.0%)
Neutropenia (< 500/μl)	6 (100.0%)
Thrombocytopenia (< 10,000/μl)	6 (100.0%)
Documented infection	1(16.7%)

ECOG: Eastern Cooperative Oncology Group; AML: Acute myeloid leukemia; MDS: Myelodysplastic syndrome; allo-HSCT: Allogeneic hematopoietic stem cell transplantation; Haplo donor: Haploidentical donor; BuCy: High-dose busulfan and cyclophosphamide; TBI: Total body irradiation. HAAG: Homoharringtonine (H), Cytarabine (A), Aclarubicin (A), Granulocyte colony stimulating factor (G); ECAG: Etoposide (E), Cytarabine (C), Aclarubicin (A), Granulocyte colony stimulating factor (G); CRS: Cytokine release syndrome; ICANS: Immune effector cell-associated neurotoxicity syndrome

Cytokine release syndrome (CRS) and immune effector cell-associated neurotoxicity syndrome (ICANS) are well-known major adverse events that limit the clinical application of CAR-T cell therapy. In our study, five patients presented mild CRS (Grade I–II), and only one experienced grade III hepatotoxicity with elevated serum transaminase and bilirubin levels according to the Common Terminology Criteria for Adverse Events (CTCAE) v4.0. All of these events were transient and clinically manageable. All patients had pancytopenia before CAR-T-38 infusion, and neutropenia appeared to persist during CAR-T-38 therapy. The median duration of neutropenia (absolute neutrophil count (ANC) of < 500/μl) was 22 (7–35) days. The median duration of platelet recovery was 25 (18–123) days. No patients manifested neurological toxicities or experienced severe infections.

Although the sample size was small and the follow-up time was limited, our preliminary prospective study highlights that the clinical utility and safety of CAR-T-38 therapy for treating AML patients with relapse post-allo-HSCT.

## Data Availability

The datasets used and/or analyzed during the current study are available from the corresponding author on reasonable request.

## Abbreviations

AML: Acute myeloid leukemia
CAR-T: Chimeric antigen receptor T cell
CAR-T-38: CD38-CAR-T cell
Allo-HSCT: Allogeneic hematopoietic stem cell transplantation
HSCs: Hematopoietic stem cells
CR: Complete remission
CRi: CR with incomplete count recovery
NR: No response
OS: Overall survival
LFS: Leukemia-free survival
CRS: Cytokine release syndrome
ICANS: Immune effector cell-associated neurotoxicity syndrome
FCM: Multiparameter flow cytometry
BuCy: High-dose busulfan and cyclophosphamide
TBI: Total body radiation
FC: Fludarabine and cyclophosphamide
CTCAE: Common Terminology Criteria for Adverse Events
ANC: Absolute neutrophil count
